# Veritas Visu Et Mora, Falsa Festinatione Et Incertis Valescunt

**Published:** 1872-09

**Authors:** 


### VERITAS VISU ET MORA, FALSA FESTINATIONE ET INCERTIS VALESCUNT.

*To the Editor of the Richmond & Louisville Medical Journal:*

In the March number of your journal you indited a squib entitled "Quackery in Medical Journals," in which you took the ground that the publication of commendatory letters in a medical journal was charlantry. This, although THE COMPANION was not named, was intended as a reflection upon it, and its management. This was a question of taste solely, and while it did not justify the epithet of charlantry—as it is almost an universal custom of the press, both literary, political and medical, as has been shown—which you so ungenerously applied to it, still as the COMPANION was not named in your article, it received no reply. Not content with this, and evidently with the desire to provoke a controversy and to bring unmerited reproach upon our Journal, you in your next number, came out in a most vindictive and vituperative denunciation of the COMPANION, charging among other things, that the editors had violated "good taste, decorum, ethics and propriety." Thus personated and abused, the COMPANION could no longer evade a controversy you sought to thrust upon it, and your vindictive article was replied to in a manner which placed you, upon your own assumption, as a violator of "good taste, decorum, ethics and propriety." In that article you were challenged upon your self-chosen ground, and the demand was made upon you to show, by the law, wherein ethics had been violated by the COMPANION. This demand you *evaded*, and your refusal to appeal to the ethics demonstrated the reckless absurdity of your charges. Finding that the ethics would not sustain you, and pressed to the wall by your own folly, you sought to escape from the difficulty by *diverting* the line of argument from an ethical to a personal one, in a crusade upon the private and professional character of the writer. You thought it easier and safer to succeed in your selfish scheme to destroy the COMPANION by making war upon my good name and reputation. Your first movement in that direction was to publish (which I have every reason to believe was furnished you by your *new*-found allies in Georgia) a review of a pamphlet without signature, but fathered by the Westmorelands. This pamphlet which you considered a God-send in your dilemma, had been circulated twelve months before your review, and upon which, with other pamphlets, you had expressed the unqualified opinion that "the Faculty of the Atlanta Medical College" should be "justly censured" by the profession. Enraged by the truthful exposure of your inconsistencies, in our reply to your strictures, you proceeded to "eat up your words" and to swallow without nausea, the slanderous statements contained in that pamphlet against the honor and integrity of almost the entire profession of this State—in order to reach one who had, in self-defense, exhibited the bloated egotism and self-inflated pretentions of the editor of "the (so-called) largest medical monthly in America." With this feeling, prompted by your ill-chosen advisors, you attempted a review of that pamphlet, throwing aside all the facts upon which you had, some months before, based your opinion that these parties were justly entitled to the deserved "censure" of yourself and the profession. In reply to this so-called review you were clearly shown the mistakes contained therein and that the "chief editor" of the COMPANION had been fully sustained, by the Board of Trustees of the Atlanta Medical College, the Fulton County Medical Society, the State Association, and the so-called charges themselves had been withdrawn by the bogus Faculty in a document addressed by them to the Trustees of the College.

At this juncture, driven to the wall the second time, your interested allies of Atlanta no doubt furnished you with the celebrated Memorial—a document the State Association declared to be false and slanderous and the publication and circulation of which by them, had received the highest penalty of that body, in expulsion—the justice of which action having been fully admitted by them at the last meeting of the Association at Columbus, by an apology and a virtual acknowledgement of falsehood on their part, as construed by those who accepted the settlement.

So, the ethical gladiator of the medical press, hugs to his bosom a document, the authors of which, for publishing, were publicly denounced as slanderers by the official action of the State Association, and in consequence thereof were expelled from that body, as the proceedings will show. This Memorial shall not consume my time here—as, by the advice of friends, I will allow you and your allies the opportunity of proving the charges therein contained against me. As stated, in our reply, a letter was published in which the charges against the writer were proposed to be withdrawn by the Faculty, and the proposition made to the Trustees by them to confer an Emeritus Professorship upon him, signed by "A. Means, Chairman of the Faculty of the Atlanta Medical College."

Of this letter I knew nothing, until it had been read at a meeting of the Trustees and rejected by them, as a cunning scheme to secure the recognition of the Board by stamping falsehood upon their previous acts. There could be no compliment to any man by a tender of a professorship from such hands—from a bogus Faculty of a College pronounced irregular by the Association, and from which body they were "expelled" as unworthy of membership; and at a meeting at which I was not present. And even now, although reduced by discipline, to a quasi-respectability, a number of the Trustees do not hesitate to affirm that it (the College) is a disgrace to the profession. And I think the facts which will be brought to light by this crusade of yours upon me, will "crop, mark and stamp" it so indelibly that it will receive the "just" and righteous "censure" of the profession as it did of yours one year ago.

In your September number you published the following letter:

ATLANTA, GA., July 19, 1872.

*Dr. E. S. Gaillard:*

DEAR SIR.—In the "Georgia Medical Companion" of June, 1872, pages 375–6, is published a paper purporting to be the proceedings of the Faculty of the Atlanta Medical College; the paper is without date, and addressed to the President of the

Board of Trustees of said College, and purports to have been signed by Dr. A. Means, as Chairman of said Faculty. Such proceedings were never had by said Faculty, with our knowledge, consent or approval; nor is there a record of such proceedings upon the minutes of the Faculty.

Of the members of the Faculty mentioned in said paper, we three are the only ones now present in the city of Atlanta; Dr. O'Keefe is deceased, Dr. Miller is temporarily absent, and Dr. Hillyer is a resident of Rome, Ga. Dr. Means's letter, herewith accompanying, speaks for him.

Respectfully, W. F. WESTMORELAND, M. D.,
J. G. WESTMORELAND, M. D.,
S. H. STOUT, M. D.,
Resigned in February, 1867.

In addition to this you copy some extracts from a letter of A. Means, in which he asserts—

"I have no recollection that this subject was ever presented to the Faculty in session, and cannot, therefore, recall any action of that body, officially, authorizing me to make such a proposition." And that he has "no recollection that the Faculty officially tendered, through myself or any one else, those terms."

Upon the above letter, endorsed by Dr. Means, you are pleased to say that "it is melancholy that any member of the medical profession could be guilty of so infamous a fraud upon the public, but when the guilty and convicted imposter proves to be one who is always prating of ethics and propriety, nothing less than the evidence here given could make the perpetration of such a fraud creditable." The only excuse—if excuse were possible—for so rash a verdict, can alone be found in the fact that you endorse a body of men of whom you are pleased to say "whose records, acts and purposes" you are "profoundly ignorant." While I must admit that the manner in which you used the extracts from Dr. Means' letter, justifies you in making him an endorser of the above charge against me, yet, knowing him as I do, and believing that he would not intentionally and willfully slander any one, I bespeak for him another hearing.

Before the September number of your journal had been issued, I had discovered by your remarks in your previous number that the authenticity of the letter of the bogus Faculty in which they attempted, through the Trustees, to confer upon me the position of "Emeritus Professor," and at the same time, as a bid in a new form for pardon, admitted that the charges against me had no truth in them—was denied by them. I, therefore, at once addressed a letter to Dr. C. L. Redwine, the Secretary of the Board, who had placed the Faculty's letter in the hands of a Committee of the Fulton County Medical Society, who, under the direction of that Society published it in a pamphlet, giving a history (from the records) of the controversy between the old Board of Trustees and this bogus Faculty of the College; also, with the Fulton County Medical Society and the Georgia Medical Association.

This letter was printed in the pamphlet from the original document as sent to the Trustees and the proof corrected by it by the committee. The one that appeared in the June number of the COMPANION is an exact copy of the one in the pamphlet. Therefore it is an exact copy of the original *official* document.

And it is worthy of note that this pamphlet was issued in April, 1871, containing this letter; was read by the profession generally, and yet it was unchallenged as a true official document. The decease of Dr. O'Keefe since that time—who was Secretary of the Faculty—may, at this late date, account for the denial of its official character, and he, if living, would doubtless testify that he delivered it to the Trustees by request of the Faculty.

ATLANTA, GEORGIA, August 21st, 1872.

*Dr. C. L. Redwine, Secretary Board of Trustees, Atlanta Medical College.*

DEAR SIR: In the June number of the GEORGIA MEDICAL COMPANION, there appeared an article in reply to Dr. Gaillard, of the *Richmond and Louisville Medical Journal*, who has thought proper to voluntarily resurrect certain charges long since refuted, against my professional record and character, in the following statement, to-wit: "The Faculty of the Atlanta Medical College have admitted the falsity of the charges themselves, in that, after the charges were made by which our reviewer asserts the "Chief Editor" was stained, the Faculty did, upon their own motion, propose to rescind all action heretofore had against him and tendered him, through the Trustees, the position of Emeritus Professor, as will be seen from the following:

*To the President of the Board of Trustees of the Atlanta Medical College:*

The Faculty of the Institution under your supervision being earnestly desirous of terminating amicably the difference existing between them and the Trustees, without further litigation, beg leave to submit, through you to the Board, that you represent the following basis of settlement, viz:

1st. That the origihal charter of the College and the amendment thereto, be superseceded by a new charter, to be obtained as early as practicable, which shall be mutually acceptable to the Trustees and Faculty.

2d. That until such charter is obtained, the Faculty shall have the power of nominating proper persons to fill all vacancies which may occur, and the Trustees the power of approving or rejecting such nominations.

3d. That the Faculty shall consist, until altered by future action of the Faculty and Trustees, of seven regular Professors and one Emeritus Professor, as follows, viz:

Thomas S. Powell, M. D., Emeritus Professor.

A. Means, M. D., Professor of Medical and General Chemistry.

D. C. O'Keefe, M. D., Professor of Theory and Practice of Medicine.

W. F. Westmoreland, M. D., Professor of Principles and Practice of Surgery.

H. V. M. Miller. M. D., Professor of Obstetrics and Diseases of Women and Children.

Ebin Hillyer, M. D., Professor of Institutes of Medicine.

S. H. Stout, M. D., Professor of Anatomy.

J. G. Westmoreland, M. D., Professor of Materia Medica and Therapeutics.

4th. As a part of this settlement, it is understood that the Faculty rescind all action heretofore had in relation to Professor T. S Powell.

A. Means,
Chairman Faculty Atlanta Medical College.

The *Richmond and Louisville Medical Journal*, for August, contained the following extraordinary statement of its editor, Dr. Gaillard, to-wit:

"One of the Faculty states that the records show why and when Powell was expelled, but there is no record of his reinstatement, or of the Faculty's action publicly claimed."

If the above extract or statement means anything at all, it means to deny the fact that there ever was any such letter emanating from the Faculty, and that Dr. Gaillard has in hand the proof to the contrary, in the person of "one of the Faculty" above referred to. In other words, it charges that the letter purporting to be addressed by A. Means, Chairman of Faculty, Atlanta Medical College, to the President of the Board of Trustees, in which it was proposed to make me "Emeritus Professor," is a forgery. It is very clear that its author intended to make the impression, secretly, upon the mind of Dr. Gaillard, that he nor his colleagues had ever admitted the falsity of the charges by which the chief editor of the COMPANION was stained. That he nor they never sactioned any proposition to rescind all action heretofore had against him. That no proposition was ever made by the Faculty or approved by him, to make him Emeritus Professor.

The language of his letter to Dr. Gaillard, as shown by the above statements made by Dr. G—, warrants the conclusions and demands of me proof of the truth of my statements made in the June number of the GEORGIA MEDICAL COMPANION.

Now sir, as you were Secretary of the Board of Trustees at the time this letter signed A. Means was received by the Trustees, and still hold the same office, you are in a position to know the facts. I therefore beg leave to propound the following questions:

1st. Is not the letter embraced in this communication which is addressed to the President of the Board of Trustees and signed by A. Means, Chairman of the Faculty, a true copy of the original?

2d. Was not the original letter sent into the Board of Trustees by the then acting Faculty as an official document?

3d. Was the original letter recorded on the minutes of the Board? If not, why?

I am, sir, yours, respectfully,

THOMAS S. POWELL.

ATLANTA, GEORGIA, August 22d, 1872.

*Dr. Thomas S. Powell, Atlanta, Ga.*

DEAR SIR: In reply to your letter of the 21st inst., I have to state that at the time the letter in question was received from the then acting Faculty of the Atlanta Medical College, I was not Secretary of the Board of Trustees, but simply a member thereof, and not being able, at this time, to lay my hand upon said communication, I can only say, in answer to your first question, that I believe the letter published to be an exact copy of the original communication which came from the Faculty. I remember, in the basis of settlement offered by the Faculty, a proposition was made by them to constitute you "Emeritus Professor."

In reply to your second question, I answer, that the communication from the Faculty was officially signed.

In reply to your third question, I would state that said communication was not recorded in the minutes of the Trustees, for the reason, as I understood it, that the Trustees did not recognize the then acting Faculty as a legally organized body, and having repeatedly refused to recognize the right of the Faculty to either fill or vacate chairs in their body, and having, by resolution, (as the minutes will show) recognized yourself as the legal Professor of Obstetrics, and requested that you would not resign said chair, they could not stultify their action by accepting such a basis of settlement. The communication in question was therefore laid upon the table. I recollect furnishing the original communication from the Faculty to the Fulton County Medical Society, and on its being returned, I have, doubtless, mislaid it. The fact, however, that such a communication was received by the Trustees, from the Faculty, is indisputable, and I presume would readily be admitted.

With feelings of personal regard, I remain, very respectfully, yours, C. L. Redwine,

Sec'y Board of Trustees, Atlanta Medical College.

The following gentlemen testify to the authenticity of the Faculty letter. It is proper to state that they have done so without solicitation on my part:

I indorse the above statements as made in the letter of Dr. Redwine to Dr. Powell, this September 30th, 1872.

Joseph Thompson,

President Board of Trustees, Atlanta Medical College.

I indorse the above statements as they are made by Dr. Redwine, this 30th of September, 1872.

D. F. Hammond,

Former Trustee Atlanta Medical College.

I remember that the communication referred to by Dr. C. L. Redwine, was received by the Trustees, proposing to make Dr. Thomas S. Powell Emeritus Professor in the Atlanta Medical College. October 2d, 1872.

Jared Irwin Whitaker, former Trustee.

I remember that there was a communication addressed to and received by the Board of Trustees of the Atlanta Medical College and I believe the above to be a true copy of the same. October 1st, 1872. William Ezzard, Trustee.

I remember that a communication signed A. Means, was received by the Board of Trustees of the Atlanta Medical College, in which it was proposed, amongst other things, to make Dr. Thos. S. Powell an Emeritus Professor in said College.

JOHN COLLIER,
Former Trustee and Sec'y of the Board.

October 4th, 1872.

ATLANTA, GEORGIA, Oct. 2d, 1872.

I remember distinctly having seen a letter addressed by Dr. A. Means, Chairman of Faculty, to the Board of Trustees of Atlanta Medical College, proposing to make Dr. Thomas S. Powell Emeritus Professor in said College. CHAS. PINCKNEY, M. D.

ATLANTA, GEORGIA, August 25th, 1872.

The letter which appears in the above correspondence, signed "A. Means, Chairman of Faculty Atlanta Medical College," in which the Faculty of that College sought to confer an Emeritus Professorship, through the Board of Trustees, upon Dr. T. S. Powell, was placed in our hands by Dr. C. L. Redwine, and is an exact and true copy of the letter so placed in our hands and signed "A. Means, Chairman of Faculty, Atlanta Medical College."

W. T. GOLDSMITH,, M. D.
J. J. KNOTT, M. D.,
E. J. ROACH, M. D.,
Committee Fulton Co., Medical Society.

Thus it will be seen that three members of the old faculty endorsed by A. Means, repudiates this letter, thereby making a direct issue of veracity between themselves and members of the Board of Trustees.

You have, without investigation, pre-judged this matter, and with your usual adroitness have attempted to place the issue between the Faculty and myself—endorsing their statements in the case. Quoting Dr. Gaillard upon himself, I do not hesitate to say that "it is melancholy that any member of the medical profession could be guilty of so infamous an act." The truth is—as all honest men will see, and which all honorable men would have, at least, sought to investigate before resorting to slanderous invectives—that the Faculty and Trustees are alone concerned in a question of veracity in the matter.

I am ready to admit, with you, but from far different motives, that a "great fraud" has been perpetrated—either by the Trustees or the Faculty, and by one of these parties upon our respective readers. As I have endorsed the statements of the Trustees, and you by renouncing your former opinions, have endorsed the bogus Faculty, our readers have the right to demand who has perpetrated "so infamous a fraud." Good ethics and morals—which you may deny in this case, but not in others—teach, that to deceive, though no untruthful words be spoken, is a *lie*. To *lie*, they also teach, and which you are wont to proclaim, is dishonorable—so dishonorable that disgrace should be forever stamped upon such a character. To be deceived by falsehood is not always dishonorable, but it is made so by endorsing it. You cannot fail to discover that either the Trustees or the signers of the letter published by you, and given verbatim above, are guilty of an "infamous fraud," and common honesty would dictate that you retract your epithets used against me, when other parties so eminently deserve them. If the facts prove the Trustees have lied, I am implicated to the extent that I published and endorsed their lie. On the other hand, if the Faculty have been guilty of the "infamous fraud" you have endorsed and published it to the injury of the Trustees. If the Trustees, by the facts, should be branded with falsehood, I for one will denounce them. Should the Faculty fail to prove themselves clear, you may still endorse them; but should you do so, you will again do violence to your record should you fail, to use your own words "to cancel publicly and promptly" so "infamous a fraud." If you are not entirely lost to the instincts of a gentleman you will own that you have done me injustice, and you will at once place the issue between the true parties. I will unite with you in any honest effort you may make in denouncing "imposters," but you cannot escape by sheltering yourself behind a convenient letter written by men whose statements have been pronounced false by the Board of Trustees, the Fulton County Medical Society, and the State Association—from which Body they were expelled, and by which Body they are said to be restored by recantation and the acknowledgment that their statements in the Memorial, in regard to the Association, were false.

You see the justice of this. If I had to rely upon the statements of your enemies in any portion of the country, as contained in pamphlets, etc., to meet you in argument, on any subject, I should abandon so convenient a route if shown to be unworthy of respect or confidence. I could no longer encourage such means unless I had some dirty end to subserve, which if true, should receive the merited contempt and scorn of every honest man.

With me, personally, it matters not what course you may choose in this matter. If you desire to blacken your record, in order to gratify your vindictiveness by refusing (to use your language) "to cancel publicly and promptly" your mistake and "to affiliate with those who you must believe, by the evidence furnished in this letter, have misrepresented the facts, you can so elect. In a warfare of this kind you can gain no honor or succeed in damaging one whose only fault is that he, in part, edits a medical journal which we have reason to believe, you desire to suppress.

I have no taste for personal controversies. Nothing is more averse to my feelings and nature. My object in life has been to aim at the accomplishment of some good in all I say and do, and hence it will be seen that I have never attempted to divert you or our readers from the issue of your own choosing, but offered to discuss them with you, logically and respectfully upon ethical principles. If I believed that even any individual good could be accomplished, I would willingly discuss with you the subjects of *fraud* and *slander.* It is not asserting too much to say that no human power can adequately portray the horried outlines of these crimes against heaven and human society. To truthfully prove a man guilty of them should forever brand him with the mark of infamy. If I had been guilty of fraud and forgery, as you and your allies have attempted to prove, I should deservedly merit the scorn and contempt of every honorable man. If guilty, its damnable stain ought to exile me from the society of the good and virtuous, and consign me to that infamy which a character so corrupt should receive from God and righteous men.

If, however, as the facts will prove, I have been grossly slandered, and that the slanderers have been guilty of the crime of fraud and forgery upon honorable men in order to shield their acts; and the worse crime of attempting to clothe an innocent party with their fraud, for his ruin, no language mortals can employ can fitly depict the devilish spirit by which they are actuated and the depravity by which they are moved.

Nor can one be far behind these unfortunate creatures preying upon the good names of their fellows, who will knowingly aid them in perpetrating a fraud, and in slandering the bright reputations of honorable men. To be the ally of such men, in such schemes, ought to and will, as long as honor and truth survives, be esteemed the last analysis of meanness, and will bring upon one so lost to honor and manliness, the scorn and righteous indignation of God and christian men.

In conclusion, I ask you to carefully review the facts given above. The gentlemen whose names appear in testimony of the authenticity of the Faculty letter, are men of position and reputation—noted for truth and honor. All have been, and some are still, members of the Board. If such facts, established by such testimony should fail to convince you of your error and you should refuse a frank and manly acknowledgement of the injustice you have done them and myself, I can no longer—with proper respect for myself and readers—recognize your claims to that degree of respect which shall call for any notice of you in future.

THOMAS S. POWELL.

---

